# Supplementary material for: Cerebellar modulation of synaptic input to freezing-related neurons in the periaqueductal gray
Source: eLife. 2020 Mar 24;9:e54302. doi: 10.7554/eLife.54302 (PMC7124251; doi:10.7554/eLife.54302)
Supplement: Transparent reporting form [file elife-54302-transrepform.docx]

***eLife’s* transparent reporting form**

We encourage authors to provide detailed information *within their submission* to facilitate the interpretation and replication of experiments. If you have any questions, please contact us: [editorial@elifesciences.org](mailto:editorial@elifesciences.org).

**Sample-size estimation**

- You should state whether an appropriate sample size was computed when the study was being designed
- You should state the statistical method of sample size computation and any required assumptions
- If no explicit power analysis was used, you should describe how you decided what sample (replicate) size (number) to use

Please outline where this information can be found within the submission (e.g., page numbers or figure legends), or explain why this information doesn’t apply to your submission:

Owing to the limited availability of animals, we recorded from as many cells as we could in each slice from each mouse with the appropriate genotype or injection site, and aimed for at least 5 cells per experiment for in vitro data, but we used multiple lines of evidence (from different but related experiments) to confirm results and support conclusions, doubling N values.

N values for cells and mice are given throughout the manuscript after each experiment is described.

**Replicates**

- You should report how often each experiment was performed
- You should include a definition of biological versus technical replication
- The data obtained should be provided and sufficient information should be provided to indicate the number of independent biological and/or technical replicates
- If you encountered any outliers, you should describe how these were handled
- Criteria for exclusion/inclusion of data should be clearly stated
- High-throughput sequence data should be uploaded before submission, with a private link for reviewers provided (these are available from both GEO and ArrayExpress)

Please outline where this information can be found within the submission (e.g., page numbers or figure legends), or explain why this information doesn’t apply to your submission:

- How often each experiment is performed is included associated with the relevant text.
- We have only biological replication (of animals, cells, and trials, indicated throughout manuscript at relevant points); technical replication is not relevant.
- All N values have been reported in the appropriate places in the text.
- No outliers were excluded from analysis based on their deviations from expected behavior. Individual data points are included in graphs.
- All quantitative and qualitative criteria for classification and inclusion are stated in the text as the relevant results are presented: i.e., series resistance stability for inclusion
- High-throughput sequence data: N/A

**Statistical reporting**

- Statistical analysis methods should be described and justified
- Raw data should be presented in figures whenever informative to do so (typically when N per group is less than 10)
- For each experiment, you should identify the statistical tests used, exact values of N, definitions of center, methods of multiple test correction, and dispersion and precision measures (e.g., mean, median, SD, SEM, confidence intervals; and, for the major substantive results, a measure of effect size (e.g., Pearson's r, Cohen's d)
- Report exact p-values wherever possible alongside the summary statistics and 95% confidence intervals. These should be reported for all key questions and not only when the p-value is less than 0.05.
- Statistical methods are given and explained in the last paragraph of the Materials and Methods. Specifics are also reported for each experiment in the text.
- Raw data (traces and values for all cells in the population) are included for each experiment.
- Statistical tests and their relevant parameters are described in the text.
- Exact p values are reported except for p<0.00001.

Please outline where this information can be found within the submission (e.g., page numbers or figure legends), or explain why this information doesn’t apply to your submission:

(For large datasets, or papers with a very large number of statistical tests, you may upload a single table file with tests, Ns, etc., with reference to page numbers in the manuscript.)

**Additional data files (“source data”)**

- We encourage you to upload relevant additional data files, such as numerical data that are represented as a graph in a figure, or as a summary table
- Where provided, these should be in the most useful format, and they can be uploaded as “Source data” files linked to a main figure or table
- Include model definition files including the full list of parameters used
- Include code used for data analysis (e.g., R, MatLab)
- Avoid stating that data files are “available upon request”

Please indicate the figures or tables for which source data files have been provided:

None.
